# Short- and long-term health effects of job insecurity. Fixed effects panel analysis of German data

**DOI:** 10.5271/sjweh.4206

**Published:** 2025-03-01

**Authors:** Małgorzata Mikucka, Oliver Arránz Becker, Christof Wolf

**Affiliations:** 1 School of Social Sciences, Mannheim University, Mannheim, Germany.; 2 Institute of Sociology, Martin-Luther-University Halle-Wittenberg, Halle an der Salle, Germany.; 3 GESIS - Leibniz-Institute for the Social Sciences, School of Social Sciences, Mannheim University, Mannheim, Germany.

**Keywords:** risk accumulation, repeated exposure, cumulative advantage, incremental effect, scarring effect, health trajectory, precarious employment, SOEP, SF-12, affective job insecurity, work precarity

## Abstract

**Objective:**

Previous research has linked job insecurity to health deterioration. The risk accumulation model suggests that health effects of job insecurity may persist even after job security is restored, yet long-term empirical analyses are scarce. Our study evaluates the long-term effects of accumulated exposures to affective job insecurity on mental and physical health among the working-age population in Germany.

**Method:**

Using data from the German Socioeconomic Panel (12 624 individuals; 84 219 observations), we applied panel regression models with individual fixed effects to assess short- and long-term health changes associated with affective job insecurity. Job insecurity was measured by respondents’ worries about job security. Mental and physical health was recorded with the SF-12 scale.

**Results:**

Job insecurity correlated with short-term worsening in mental and physical health. However, after job insecurity ceased, health recovery was incomplete resulting in a long-term health deterioration. The long-term effects were larger among respondents who accumulated more instances of job insecurity, and showed a similar pattern for mental and physical health. An additional analysis documented stronger health effects of job insecurity among lower educated persons.

**Conclusion:**

Our study is one of the first to empirically demonstrate the negative long-term health effects of job insecurity. Our findings for a well-protected labor market like Germany’s, suggest that the health risks associated with job insecurity may be substantial and potentially underestimated by studies that focus solely on short-term effects.

Job insecurity, defined as the subjectively perceived risk of losing one’s job or a concern over losing it, consistently correlates with poor health outcomes (1–4). Stress is a key mediator, arising from the anticipated loss of financial and latent work benefits (5, 6), as well as feelings of unpredictability, hopelessness, and lack of control. Indeed, job insecurity has been shown to impact health as severely as impending dismissal or unemployment (7). Job insecurity impairs health through direct physiological stress responses (8), but also via unhealthy coping behaviors like drinking, smoking, and substance use (9). Extensive research highlights the detrimental effects of job insecurity on health, with evidence covering various aspects of both mental and physical health (1–4, 10–14).

Past research has primarily focused on the short-term effects of job insecurity, implicitly assuming that health recovers once insecurity ends. However, epidemiological and sociological models suggest that health is shaped over the long term through repeated exposures to risk factors. The risk accumulation model proposes that the health effects of risk factors like job insecurity can build up over an individual’s life (15). Similarly, theories of cumulative (dis)advantage imply that risks and resources accumulate over time to produce diverging age trajectories of health (16, 17). This suggests that the effects of job insecurity can persist long after it ends, with both current and past exposures contributing incrementally to health outcomes.

The evidence on long-term health effects of job insecurity is scarce. Donnelly (18) demonstrated that chronic midlife exposure to precarious work, including job insecurity, shaped health trajectories after age 65, increasing chronic conditions and functional limitations. Another group of studies focused on the duration of job insecurity, finding more severe health effects of persistent (or “chronic”) insecurity compared to short-term insecurity (10–13). Unfortunately, these studies did not differentiate between short- and long-term effects, considered only a few observations per individual, and studied final health rather than health trajectories, limiting their suitability to analyze long-term health effects. Another stream of research provided suggestive evidence, linking job insecurity with reduced subjective well-being (19, 20), and documenting the scarring effects of unemployment on careers (21, 22) and health (23–26).

Overall, while previous studies hint at mechanisms that could produce the long-term health effects of accumulated job insecurity, direct empirical evidence is lacking. We aim to address this gap. Using biennial data spanning 9–19 years, we provide a robust longitudinal framework that enables us to quantify the exposure to job insecurity, track health trajectories rather than consider health outcomes at a single time point, and differentiate between short- and long-term effects.

## Methods

### Study sample

Our study used data from the German Socio-Economic Panel (SOEP, version 38), an extensive annual survey encompassing nearly 11 000 households and 30 000 individuals each year (27). It is representative of the German resident population and includes specific subsamples, such as migrants, high-income households, and specific family types. Key to our research were the SOEP’s biennially recorded measures of physical and mental health as captured by the SF-12 scale (available for 2002–2020), and data on employment situations recorded yearly. Ethics approval was not required for this study as it involved the use of publicly available data that did not include personal identifiers.

Our initial sample included all observations from the waves when health outcomes were recorded (138 221 respondents and 459 072 observations). In the first step, we selected respondents at risk of job insecurity, ie, persons aged 18–65, who had entered the labor market and had not retired (we excluded observations from the two years preceding retirement as people may be more likely to accept insecurity in order to bridge to retirement), keeping 53 491 respondents and 192 270 observations. Second, to ensure a sufficient observation window for analyzing long-term effects, we retained respondents observed over a minimum of 9 years (at least 5 observations for health), keeping 16 058 respondents and 108 375 observations. Finally, list-wise exclusion of missing responses further reduced the sample to 12 624 respondents and 84 219 observations. [Overall, 11.9% of observations had some missing values, with the highest shares for job insecurity (11.1%), employment status (9.2%), health (9.3%), and job change (9.2%)].

### Study variables

We ran separate analyses for physical and mental health, as recorded by the SF-12 scale (27, 28), which measures self-reported health-related quality of life based on general health, physical and social functioning, mental health, bodily pain, physical and emotional restrictions on social role accomplishment, vitality, and health-related restrictions on social contacts. We derived scores for mental and physical health from confirmatory factor analysis with correlated factors (wave-specific correlations 0.76–0.80). We rescaled the dependent variables to a 0–100 range to simplify coefficient interpretation, with higher values indicating better health.

We measured affective job insecurity using a dichotomous variable based on the question: “How concerned are you about the following issues? (…) If you are employed: Your job security.” Employed respondents who reported being “very” or “somewhat concerned” were coded as 1, while those “not at all concerned” were coded as 0. We also constructed a cumulative measure that counted, for each time point, current and past occurrences of affective job insecurity. This measure started at 0 or 1 for an individual’s first observation and increased by 1 with each subsequent observation indicating job insecurity. It encompassed data from all waves, whether or not health outcomes were recorded, and did not increase during periods of secure employment, economic inactivity, unemployment, or when employment data were missing. (Whereas our main analysis did not differentiate the health effects of severe and mild insecurity, we explored this distinction in an additional analysis: see ‘Sensitivity Analyses’ section and table S7 in supplementary material https://www.sjweh.fi/article/4206 .)

Leaving insecure employment can trigger health recovery, either immediately or after a delay. To ensure that our estimates of the long-term effects of job insecurity did not reflect the delayed recovery, we included dummy variables marking the first, second, and third year after leaving insecure employment.

We chose continuously affectively secure employees (ie, those who, over the observation period, never experienced job insecurity, unemployment, or inactivity) as our reference category; therefore, we controlled for periods of unemployment and economic inactivity (based on Labor Force Survey criteria) coded as dichotomous variables. During these periods, the measure of job insecurity took the value of 0. We also controlled for accumulated unemployment and inactivity, considering these as potential confounders in the relationship between accumulated job insecurity and health. On the one hand, accumulated job insecurity is likely to correlate with accumulated unemployment and inactivity; on the other hand, prolonged unemployment had been shown to impair health (23–26).

Age was a likely confounder in our analysis, because both health and the risk of job insecurity differ with age (14, 29), we therefore controlled for linear and quadratic effects of age (centered at 40 years). Another possible confounder was socioeconomic status, because both the rate of health worsening with age (16, 30) and the risk of job insecurity (14, 31) tend to vary with socioeconomic position. To address this, we included an interaction of years of schooling (based on the highest education level reported by respondents, thus making this variable time-invariant) with age to control for socioeconomic differences in the baseline rate of health erosion. Similarly, we accounted for variations in health erosion rates between genders and across cohorts. Additionally, to account for the possibility that job changes confound the results by affecting future health and job insecurity, we controlled for self-declared job changes occurring over the previous two years.

Finally, acknowledging that labor market regulations and economic conditions likely influenced both health outcomes and employment patterns, we included year dummies. Specifically, we controlled for the impact of the Hartz reforms, which extended the low-pay sector and increased job insecurity in Germany, by introducing a dummy for the period before 2005. We also accounted for economic recession periods by including dummies for 2002–2003, 2009–2010, and 2020 (32) – the latter also capturing the health effects of the COVID-19 pandemic. The outcome variables and all time-varying predictors were measured in each time-point available for a given respondent. Table 1 provides an overview of all variables used in the analysis.

**Table 1:** Characteristics of the study population. SOEP, 2002–2020.[SD=standard deviation; Min=minimum; Max=maximum]

Variable			Mean	SD	N	%	Min	Max
Time-varying variables (N= 84 219 observations)								
	Mental health (0–100)		69.30	14.89			0	100
	Physical health (0–100)		68.86	17.07			0	100
	Job insecurity							
		Current			33 231	39.46	0	1
		Cumulative exposure	3.07	3.53			0	19
		Recovery year 1			9236	10.97	0	1
		Recovery year 2			5258	6.24	0	1
		Recovery year 3			3386	4.02	0	1
	Unemployment							
		Current			4247	5.04	0	1
		Cumulative exposure	0.37	1.23			0	19
		Recovery year 1			1733	2.06	0	1
		Recovery year 2			1407	1.67	0	1
		Recovery year 3			1105	1.31	0	1
	Inactivity							
		Current			7998	9.50	0	1
		Cumulative exposure	0.72	1.82			0	19
		Recovery year 1			2453	2.91	0	1
		Recovery year 2			2315	2.75	0	1
		Recovery year 3			1791	2.13	0	1
	Age		44.50	9.30			18	65
	Before Hartz reforms (before 2005)				13 446	15.97	0	1
	Recession 2002–3				6545	7.77	0	1
	Recession 2009–10				10 531	12.50	0	1
	COVID-19 (2020)				6811	8.09	0	1
	Reference years				60 344	71.65	0	1
Time-invariant variables (N=12 624 respondents)								
	Years of schooling		12.81	2.73			7	18
	Woman				7005	55.48	0	1
	Birth year (cohort)		1967.06	9.62			1945	1994

### Data analyses

To assess short- and long-term health changes associated with job insecurity, we used fixed effects (FE) panel models with standard errors clustered on individuals. FE models account for unobserved time-invariant individual differences, such as baseline health, personality traits, education, gender, or cohort of birth (33, 34). By including interactions of age with education, gender, and cohort, we allowed health ageing trajectories to vary with sociodemographic characteristics, thereby relaxing the “parallel trends” assumption. Our FE model is outlined in equation (1) (i=individual, t=time):


(1) $${Health}_{it}=\alpha +\beta_{1}{INSECURE}_{it}+\beta_{2}{CUMUL.INSECURITY}_{it}+\beta_{3}{CUMUL.INSECURITY}_{it}^{2}+B_{4}{Recovery}_{it}+\beta_{5}{Age}_{it}+\beta_{6}{Edu}_{i}\times {Age}_{it}+\beta_{7}{Woman}_{i}\times {Age}_{it}+\beta_{8}{Cohort}_{i}\times {Age}_{it}+B_{9}{CONTROL}_{it}+\;u_{i}+\varepsilon_{it}$$

In this equation, time-varying individual health (Health_it_) is regressed on current job insecurity (coefficient β_1_) and accumulated exposure to insecurity (β_2_ and β_3_ capture the effects of linear and quadratic terms). Health and job insecurity are observed over time, therefore the short-term effect of job insecurity (β_1_) refers to health shifts associated with the onset and cessation of insecurity. The long-term effects (β_2_ and β_3_) allow each additional exposure to affective insecurity to have lasting health consequences. The recovery dummies (vector of coefficients B_4_) account for short-term health shifts following the end of job insecurity, allowing for delayed recovery. The model also accounts for the age-related health deterioration (β_5_) and differences in health deterioration across groups (β_6_-β_8_). All the time-varying variables are observed at the same time at each wave (t). The variables “education”, “woman”, and “cohort” are time-invariant, therefore their main effects are captured by individual-specific intercepts (u_i_) and not estimated separately. B_9_ is a vector of coefficients of control variables, including unemployment, inactivity, and year dummies. Finally, ε_it_ is the time-varying residual. Analyses were performed using Stata Statistical Software, Release 14 (StataCorp LP, College Station, TX, USA).

## Results

### Descriptive results

Affective job insecurity was frequent in our sample, with 80% of participants experiencing it at least once, and 39% of all observations reflecting some degree of worry about job security. Half of the respondents accumulated at least four instances of job insecurity, whereas 5% experienced it ≥14 times. Unemployment and economic inactivity were less common, affecting, respectively, 20% and 32% of participants at least once over the observation period, with only a small fraction experiencing them multiple times. (For details, see supplementary table S1.) About 10% of respondents in our sample were continuously affectively secure, ie, they experienced no job insecurity, no inactivity and no unemployment over the observation span.

### Multivariate analysis

Table 2 shows the results of FE estimation for mental and physical health, where our focus lies on the coefficients related to job insecurity. The negative coefficients of current job insecurity indicate that both mental and physical health worsened during periods of affective job insecurity. The indicators of recovery were not statistically significant, meaning that health recovery post-job insecurity was immediate rather than gradual or delayed. The negative long-term effect of accumulated exposure to job insecurity means that each instance of job insecurity predicted an incremental long-term health worsening. The statistically significant and positive effect of squared cumulative exposure means that these long-term effects were somewhat more pronounced for initial instances of job insecurity, diminishing with each additional period of insecurity.

**Table 2:** Short- and long-term effects of job insecurity on mental and physical health (0-100). Fixed effect estimation with standard errors clustered on individuals. SOEP, 2002–2020, N= 84 219 observations and N=12 624 respondents.[B=unstandardized coefficients; CI=confidence intervals]

		Mental health (0–100)			Physical health (0–100)	
		B	95% CI		B	95% CI
Job insecurity						
	Current	-1.92	-2.23– -1.60		-1.57	-1.91– -1.22
	Cumulative exposure	-0.39	-0.52– -0.26		-0.47	-0.62– -0.33
	Cumulative exposure squared	0.02	0.01–0.02		0.02	0.01–0.03
	Recovery year 1	-0.07	-0.41–0.28		-0.11	-0.49–0.27
	Recovery year 2	-0.17	-0.53–0.20		-0.11	-0.52–0.30
	Recovery year 3	0.13	-0.30–0.56		0.17	-0.30–0.63
Unemployment						
	Current	-4.62	-5.34– -3.91		-3.18	-3.94– -2.41
	Cumulative exposure	-0.57	-0.94– -0.20		-0.91	-1.28– -0.53
	Cumulative exposure squared	0.02	-0.02–0.05		0.03	-0.00–0.06
	Recovery year 1	0.07	-0.67–0.82		0.32	-0.46–1.10
	Recovery year 2	0.04	-0.72–0.80		0.31	-0.51–1.12
	Recovery year 3	-0.07	-0.88–0.74		0.72	-0.12–1.57
Inactivity						
	Current	-2.14	-2.65– -1.62		-2.15	-2.72– -1.59
	Cumulative exposure	-0.17	-0.41–0.08		-0.02	-0.28–0.23
	Cumulative exposure squared	0.00	-0.02–0.02		-0.00	-0.02–0.02
	Recovery year 1	0.03	-0.55–0.62		-0.39	-1.02–0.25
	Recovery year 2	-0.76	-1.32– -0.19		-0.67	-1.26– -0.07
	Recovery year 3	-0.74	-1.37– -0.11		-0.10	-0.72–0.52
Age (centered at 40, per 10 years)		-3.32	-4.44– -2.21		-6.04	-7.26– -4.82
Age × age		1.45	0.75–2.14		1.03	0.27–1.79
Age × years of schooling (centered at 12 years)		0.18	0.10–0.26		0.38	0.30–0.47
Age × woman		-0.31	-0.77–0.15		0.08	-0.42–0.58
Age × cohort (centered at 1965, per 10 years)		3.20	1.84–4.55		3.07	1.58–4.56
Job change		0.93	0.56–1.31		1.14	0.73–1.56
Before Hartz reforms (before 2005)		-0.62	-1.05– -0.19		-0.53	-0.98– -0.07
Recession 2002–3		-1.77	-2.20– -1.34		-2.05	-2.50– -1.59
Recession 2009–10		-0.05	-0.28–0.18		0.04	-0.21–0.30
COVID-19 (2020)		-1.65	-2.09– -1.20		-0.32	-0.80–0.16
Constant		73.50	72.76–74.25		74.19	73.38–75.00
R-squared within		0.018			0.041	

**Figure 1:**
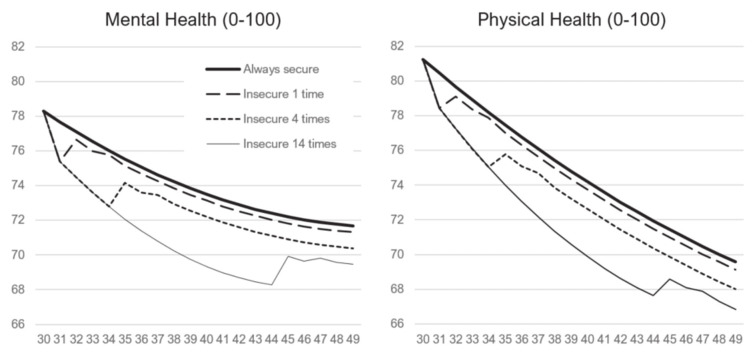
Predicted health trajectories under four scenarios: (i) continuously affectively secure employees, (ii) individuals insecure at age 31 (single exposure), (iii) individuals insecure aged 31–34 (four exposures), and (iv) individuals insecure aged 31–44 (14 exposures to affective job insecurity). The predictions for men born in 1965 are based on the models presented in table 2, and assume 12 years of education and no experience of unemployment nor inactivity. The horizontal axis refers to age.

Figure 1 illustrates these patterns and the effect sizes by showing stylized predicted health trajectories for individuals who experienced job insecurity 0, 1, 4, and 14 times over a 20-year period. For continuously affectively secure employees, the graph shows the typical age-related health worsening. Compared to this group, everyone experiencing job insecurity faced additional health decreases. Those who experienced job insecurity once showed a short-term reduction in health, which recovered immediately after the insecurity ended. However, the recovery was incomplete: a single exposure to job insecurity left a long-term negative effect of about 20–30% of the respective short-term effects (20% for mental health and 29% for physical health; for these and following predictions see supplementary table S12). Individuals exposed to job insecurity 4 times experienced a short-term health decline during the period of insecurity, followed by partial recovery. Here, the long-term effect was larger, corresponding to 68% of the short-term effect for mental health and 101% for physical health. Finally, the long-term effect of accumulating 14 instances of job insecurity corresponded to 115% of the short-term effect for mental health and 175% for physical health. Comparison of the effects of 4 and 14 exposures shows the diminishing marginal effects of subsequent exposures to insecurity, indicated in table 2 by the positive quadratic effect of accumulated exposure. The first 4 exposures predicted a long-term health reduction in mental health of 1.30 points, averaging 0.32 point per year, whereas the subsequent 10 exposures predicted only an additional 0.91 point reduction, ie, 0.09 point per year on average. The results for physical health showed a similar pattern.

Like job insecurity, unemployment also exhibited both short- and long-term health effects, with no evidence of delayed or gradual recovery. In contrast, inactivity was only associated with short-term health worsening and did not appear to have a long-term effect. However, negative health outcomes persisted over the three years after inactivity ceased, as indicated by negative recovery coefficients.

The age effect comprised a negative linear coefficient and positive quadratic coefficient, indicating a slowdown in health decline with age. Controlling for the long-term effects of accumulated insecurity and unemployment ensured that ageing coefficients referred to continuously affectively secure employees. For this group, between ages 30 and 50 predicted mental health declined by 6.65 points and physical health declined by 12.08 points. Interactions with education, cohort, and gender inform about a slower decline among higher-educated and in younger cohorts, with no significant gender differences.

### Sensitivity analyses

*Stability of coefficients.* Including multiple interrelated effects in a single model may raise concerns about collinearity. Supplementary tables S2–S3 compare different model specifications (models with short-term effects only and with linear, but not quadratic, long-term effects) against the models presented in the main body of the paper, and they demonstrate the stability of coefficients for short-term effects, recovery, and age across models.

*Self-rated health.* Due to longer observation window (1992–2021, ie, up to 29 years) and for comparability with previous studies (eg 12, 13,), we estimated linear probability models (36) using “good” or “very good” self-rated health as the outcome (see supplementary tables S4–S5 for sample characteristics and table S6 for regression results). The analysis yielded similar results, showing a short-term health worsening during periods of insecurity followed by an immediate yet incomplete recovery. The statistically significant long-term effects, comprising linear negative effects and positive quadratic effects, predict stronger negative effects for initial exposures to insecurity and smaller marginal effects of subsequent exposures.

*Degree of worry about job security.* The SOEP differentiates between being “very” and “somewhat” worried about job security, enabling us to test dose–response effects. Severe worry about job security is a much less common experience than mild insecurity (see supplementary tables S1 and S5), affecting 41% of respondents compared to 77.5% for mild insecurity. The top 5% of respondents experienced severe insecurity six or more times. The additional analyses (see supplementary table S7) showed that severe job insecurity was associated with greater short-term health worsening than mild insecurity. Moreover, the long-term effects of accumulated exposures were also stronger for severe insecurity than for mild insecurity. This dose–response relationship held for mental, physical, and self-rated health.

*Effects heterogeneity: analyses by gender and education.* Given the possible differences by gender and education level, we estimated models for men versus women (see supplementary tables S8–S9) and higher versus lower educated (see supplementary tables S10–S11). Our results showed similar patterns for both genders, with somewhat stronger short-term effects among men and stronger long-term effects among women. Educational differences showed a clear-cut pattern, with substantially larger effects (both short- and long-term) of job insecurity among the lower-educated.

## Discussion

Our study addressed a gap in the literature by integrating the risk accumulation model and the cumulative (dis)advantage framework (15, 16) – which posit that health is shaped by long-term exposures – with research practice that often overlooks long-term effects. Our study estimated the long-term effects of accumulated affective job insecurity on the health of the German working-age population, assessing its effects on mental and physical health over a period of up to 19 years, and on self-rated health for up to 29 years. Our study is among the first to document the long-term effects of job insecurity.

Our results suggested that, although individuals largely recover from the short-term health consequences of job insecurity, each exposure to insecurity leaves a lasting negative footprint on their health. The consistency of these findings across various health outcomes (mental, physical, and self-rated health), as well as the results for mild and severe affective job insecurity, support the robustness of our conclusions. The magnitude of the long-term effects can be contextualized by comparing them to the health erosion occurring with age among continuously affectively secure employees. Translated into this metric, a single exposure to job insecurity results in an additional long-term health decline similar in magnitude to the one that affectively secure employees experience over the course of one year (see supplementary table S12). Four instances of job insecurity permanently reduce mental health by the equivalent of 3.9 years (2.6 for physical health), whereas 14 instances of insecurity permanently reduce mental health by the equivalent of 6.6 years (4.5 for physical health; see supplementary table S12). The diminishing marginal effects of subsequent exposures to job insecurity, as indicated in our model by the positive effect of squared exposures, may suggest a desensitization process, where the health effects of repeated exposures are weaker than those of earlier ones. Our study is the first to explore and discuss this pattern.

Comparing our findings to previous studies is difficult due to differences in data structure, measurement, and analytical design, as well as the limited focus on long-term effects in past research. One exception is a study that estimated that 16 past exposures to job insecurity increased chronic conditions by the equivalent of 1.38 years (18). This may be due to the study’s focus on cognitive rather than affective insecurity or because chronic conditions reflect more severe health issues than our measure, requiring more insecurity to produce a similar effect. The empirical patterns presented by our study are consistent with research documenting long-term effects of past insecurity (12, 18–20), and align with analyses reporting stronger negative effects of persistent insecurity (11, 13).

Our study presents job insecurity as a widespread problem, being reported in 39% of observations at a given point in time and by 80% of respondents at least once over a 9–19-year period. This high prevalence of affective insecurity aligns with previous research. For instance, 47% of young employees (aged 27–30) with permanent contracts and 65% with fixed-term contracts reported worry about job security (20). Cross-sectional estimates of affective insecurity in European countries range from 23–46%, with a median of 38% (37). By tracking individuals over time, we show that the proportion ever affected is much higher than the cross-sectional data suggest.

Our study found similar patterns for job insecurity and unemployment, with both showing short-term health effects, followed by an immediate but incomplete recovery, and leaving a long-term footprint after the exposure ends. However, both short- and long-term effects of unemployment were substantially stronger (around twice as high) than those of affective insecurity. Additionally, the quadratic component of long-term effects was not significant for unemployment, suggesting that the desensitization seen for job insecurity does not occur for unemployment.

Our additional analyses explored differences across educational levels and genders. Consistently with previous studies (12, 18), we found small and rather inconsistent gender differences. However, the effects were more pronounced among lower educated than among higher educated people, suggesting greater vulnerability of this group. This contrasts with earlier research looking at subjective well-being outcomes and documenting stronger effects among higher educated (20), which may reflect different mechanisms shaping health and subjective well-being.

The strength of our study lies in its longitudinal design, allowing us to observe insecurity and health trajectories over 9–19 years for physical and mental health, and 9–29 years for self-rated health. This design is crucial for estimating long-term effects, distinguishing them from short-term effects, and allowing for the control of delayed or gradual recovery processes. Moreover, by accounting for the socioeconomic gradient in health sustainability, we controlled for the potentially confounding role of socioeconomic differences for the baseline health trajectories, thus reducing the risk of an upward bias in the estimated health effects of job insecurity.

Our analysis included about 18% of the source population (9% of source respondents), raising concerns about its representativeness and validity. The consequences of selection vary by stage. Limiting the sample to working-age respondents who ever participated in the labor market (a 58% reduction) seems unproblematic as it retained everybody at risk of job insecurity. In contrast, retaining respondents observed for ≥9 years (23% of the original sample) likely introduced bias as those selected could have more stable employment, residence, and better health. Similarly, selecting observations with valid employment data might have retained those with more stable work histories. Both factors likely reduced the variance of predictors and outcomes, leading to conservative estimates. Finally, caution is needed when applying our findings to younger cohorts (eg, born in the 1990s) as these individuals were less likely to be observed for a minimum of nine years.

Our intra-individual approach using individual fixed intercepts mitigated some concerns about unobserved heterogeneity and selection effects. Nonetheless, FE methods remain vulnerable to time-varying confounders, such as objectively disadvantageous working conditions, workplace organization, or macro-level factors like regional or industry-specific unemployment rates, all of which may shape affective job insecurity and health. Indeed, past research suggested that the health effects of objective conditions are mediated by their subjective perceptions (20, 38, 39), capturing the idea that adverse conditions shape health as long as they are perceived. Analyzing the interdependencies between objective and subjective insecurity and health is beyond the scope of our study, but remains a promising path for future research.

Although past research has generally supported a causal direction from job insecurity to health rather than the reverse (1, 40), the possibility of reverse causality in our analysis cannot be entirely excluded. For instance, individuals in poorer health may be less able to change jobs when facing job insecurity, potentially leading to longer exposure. However, our study linked longer accumulation not to overall worse health, but to a stronger health decline. Nonetheless, these concerns cannot be fully addressed by our design, warranting future research using causal methods such as instrumental variables.

Another limitation is the use of self-reported data for both job insecurity and health, which may introduce bias: individuals who report worse health might also report higher insecurity, a tendency we might call “pessimism.” Our fixed effects method controls for time-invariant individual differences, including pessimism, but cannot capture the effects of changes in pessimism in response to experienced job insecurity. Future studies may explore these time-varying effects.

Another limitation is that our findings are context-specific to the German labor market, known for its substantial unemployment insurance and active labor market policies (41), which, combined with the universal health insurance, may buffer the health effects of job insecurity (42). The effects estimated under less protective systems could be stronger, but studies from other countries would be necessary to validate this hypothesis.

Our study makes theoretical and practical contributions. The theoretical contribution is to document a pattern theorized by the risk accumulation model and the cumulative (dis)advantage framework which has not been previously verified empirically: job insecurity has long-term negative effects on individual health. These findings underscore the importance of conceptualizing the time progression when theorizing the effects of events, suggesting a promising path for future studies. Conceptualizing and estimating a variety of possible effects (short- and long-term, delayed, threshold effects) may enhance our understanding of these complex relationships.

Practically, our analysis highlights long-lasting negative health effects of affective job insecurity, a particularly worrisome issue given that 80% of our working-age study population experienced some job insecurity, with half exposed to it ≥4 times. Our findings suggest that the health costs of job insecurity may be underestimated when only short-term effects are considered. The prevalence of prolonged job insecurity, likely to increase in the future with ever-more flexible labor markets, underscores the need for a broader social debate on this urgent topic.

## Supplementary material

Supplementary material
