# Supplementary material for: Short- and long-term health effects of job insecurity. Fixed effects panel analysis of German data
Source: Scand J Work Environ Health. 2025 Feb 28;51(2):68–76. doi: 10.5271/sjweh.4206 (PMC11887650; doi:10.5271/sjweh.4206)
Supplement: Supplementary material [file SJWEH-51-68-S001.pdf]

Short- and long-term health effects of job insecurity. Fixed effects panel analysis of German data[1]

by Małgorzata Mikucka, PhD,[2] Oliver Arránz Becker, academic degree, Christof Wolf, academic degree

1. *Supplementary material*
2. *Correspondence to: Malgorzata Mikucka, School of Social Sciences, Mannheim University, A5, 6, D-68159 Mannheim, Germany. [E-mail: malgorzata.mikucka@uni-mannheim.de]* https://orcid.org/0000-0002-9648-0939.

Table S1. Distribution of cumulative exposure to job insecurity, unemployment, and inactivity in the studied sample. SOEP, 2002-2020, n = 12,624 respondents.

	Job insecurity				
	Overall	Severe (“very worried”)	Mild (“somewhat worried”)	Unemployment	Inactivity
Percent who experienced none	19.56%	58.95%	22.46%	80.05%	68.31%
Accumulated exposure - 25th percentile	1	0	1	0	0
Accumulated exposure - median	4	0	3	0	0
Accumulated exposure - 75th percentile	8	1	6	0	1
Accumulated exposure - 95th percentile	14	6	11	4	7
Accumulated exposure - 99th percentile	18	10	15	9	12

Table S2. Alternative specifications of regression of mental health (0-100) on (current and accumulated) job insecurity. Fixed effect estimation with standard errors clustered on individuals. SOEP, 2002-2020, N= 84,219 observations and n = 12,624 respondents.[B – unstandardized coefficients, CI – confidence intervals]

	Model 1 Short-term effects only		Model 2 Linear long-term effects		Main model Quadratic long-term effects	
	B	95% CI	B	95% CI	B	95% CI
Job insecurity:						
Current	-2.24	-2.54– -1.93	-2.07	-2.38– -1.75	-1.92	-2.23– -1.60
Cumulative exposure			-0.14	-0.21– -0.07	-0.39	-0.52– -0.26
Cumulative exposure squared					0.02	0.01– 0.02
Recovery year 1	-0.32	-0.66– 0.01	-0.19	-0.54– 0.15	-0.07	-0.41– 0.28
Recovery year 2	-0.37	-0.74– -0.01	-0.27	-0.64– 0.09	-0.17	-0.53– 0.20
Recovery year 3	-0.04	-0.47– 0.39	0.02	-0.41– 0.45	0.13	-0.30– 0.56
Unemployment:						
Current	-4.92	-5.61– -4.22	-4.63	-5.33– -3.93	-4.62	-5.34– -3.91
Cumulative exposure			-0.43	-0.63– -0.24	-0.57	-0.94– -0.20
Cumulative exposure squared					0.02	-0.02– 0.05
Recovery year 1	-0.17	-0.88– 0.54	0.09	-0.64– 0.82	0.07	-0.67– 0.82
Recovery year 2	-0.17	-0.92– 0.58	0.03	-0.72– 0.79	0.04	-0.72– 0.80
Recovery year 3	-0.37	-1.16– 0.43	-0.11	-0.91– 0.69	-0.07	-0.88– 0.74
Inactivity:						
Current	-2.24	-2.74– -1.73	-2.13	-2.63– -1.62	-2.14	-2.65– -1.62
Cumulative exposure			-0.12	-0.25– 0.00	-0.17	-0.41– 0.08
Cumulative exposure squared					0.00	-0.02– 0.02
Recovery year 1	0.02	-0.55– 0.58	0.07	-0.51– 0.65	0.03	-0.55– 0.62
Recovery year 2	-0.75	-1.30– -0.20	-0.71	-1.27– -0.15	-0.76	-1.32– -0.19
Recovery year 3	-0.77	-1.39– -0.15	-0.73	-1.35– -0.10	-0.74	-1.37– -0.11
Age (centered at 40, per 10y)	-4.79	-5.82– -3.76	-3.68	-4.78– -2.58	-3.32	-4.44– -2.21
Age x Age	1.75	1.07– 2.44	1.59	0.90– 2.28	1.45	0.75– 2.14
Age x Years of schooling (centered at 12y)	0.26	0.18– 0.33	0.19	0.10– 0.27	0.18	0.10– 0.26
Age x Woman	-0.36	-0.79– 0.08	-0.35	-0.81– 0.11	-0.31	-0.77– 0.15
Age x Cohort (centered at 1965, per 10y)	3.67	2.33– 5.01	3.38	2.03– 4.72	3.20	1.84– 4.55
Job change	0.80	0.43– 1.18	0.85	0.48– 1.22	0.93	0.56– 1.31
Before Hartz reforms (before 2005)	-0.52	-0.95– -0.09	-0.52	-0.95– -0.09	-0.62	-1.05– -0.19
Recession 2001-3	-1.68	-2.10– -1.25	-1.65	-2.07– -1.22	-1.77	-2.20– -1.34
Recession 2009-10	-0.07	-0.30– 0.16	-0.06	-0.29– 0.17	-0.05	-0.28– 0.18
COVID-19 (year 2020)	-1.65	-2.09– -1.20	-1.65	-2.09– -1.20	-1.65	-2.09– -1.20
Constant	-0.52	-0.95– -0.09	-0.52	-0.95– -0.09	-0.62	-1.05– -0.19
R-squared within	0.017		0.018		0.018	

Table S3. Alternative specifications of regression of physical health (0-100) on (current and accumulated) job insecurity. Fixed effect estimation with standard errors clustered on individuals. SOEP, 2002-2020, N= 84,219 observations and n = 12,624 respondents. [B – unstandardized coefficients, CI – confidence intervals]

	Model 1 Short-term effects only		Model 2 Linear long-term effects		Main model Quadratic long-term effects	
	B	95% CI	B	95% CI	B	95% CI
Job insecurity:						
Current	-1.96	-2.29– -1.63	-1.75	-2.09– -1.41	-1.57	-1.91– -1.22
Cumulative exposure			-0.18	-0.25– -0.10	-0.47	-0.62– -0.33
Cumulative exposure squared					0.02	0.01– 0.03
Recovery year 1	-0.43	-0.79– -0.06	-0.26	-0.64– 0.11	-0.11	-0.49– 0.27
Recovery year 2	-0.37	-0.78– 0.04	-0.24	-0.65– 0.17	-0.11	-0.52– 0.30
Recovery year 3	-0.06	-0.52– 0.41	0.02	-0.44– 0.49	0.17	-0.30– 0.63
Unemployment:						
Current	-3.66	-4.41– -2.92	-3.26	-4.01– -2.51	-3.18	-3.94– -2.41
Cumulative exposure			-0.61	-0.82– -0.41	-0.91	-1.28– -0.53
Cumulative exposure squared					0.03	-0.00– 0.06
Recovery year 1	-0.11	-0.87– 0.65	0.27	-0.50– 1.04	0.32	-0.46– 1.10
Recovery year 2	-0.04	-0.85– 0.77	0.25	-0.56– 1.06	0.31	-0.51– 1.12
Recovery year 3	0.26	-0.58– 1.10	0.63	-0.22– 1.47	0.72	-0.12– 1.57
Inactivity:						
Current	-2.16	-2.71– -1.62	-2.09	-2.65– -1.54	-2.15	-2.72– -1.59
Cumulative exposure			-0.05	-0.19– 0.09	-0.02	-0.28– 0.23
Cumulative exposure squared					-0.00	-0.02– 0.02
Recovery year 1	-0.26	-0.88– 0.35	-0.29	-0.92– 0.33	-0.39	-1.02– 0.25
Recovery year 2	-0.56	-1.15– 0.03	-0.58	-1.17– 0.01	-0.67	-1.26– -0.07
Recovery year 3	-0.04	-0.66– 0.57	-0.05	-0.68– 0.57	-0.10	-0.72– 0.52
Age (centered at 40, per 10y)	-7.85	-8.97– -6.73	-6.45	-7.66– -5.25	-6.04	-7.26– -4.82
Age x Age	1.38	0.62– 2.13	1.19	0.44– 1.95	1.03	0.27– 1.79
Age x Years of schooling (centered at 12y)	0.48	0.40– 0.56	0.39	0.31– 0.48	0.38	0.30– 0.47
Age x Woman	0.16	-0.32– 0.63	0.05	-0.45– 0.55	0.08	-0.42– 0.58
Age x Cohort (centered at 1965, per 10y)	3.64	2.16– 5.11	3.28	1.80– 4.76	3.07	1.58– 4.56
Job change	1.00	0.59– 1.41	1.05	0.64– 1.46	1.14	0.73– 1.56
Before Hartz reforms (before 2005)	-0.40	-0.85– 0.05	-0.41	-0.86– 0.04	-0.53	-0.98– -0.07
Recession 2001-3	-1.94	-2.39– -1.50	-1.91	-2.36– -1.46	-2.05	-2.50– -1.59
Recession 2009-10	0.01	-0.24– 0.27	0.03	-0.22– 0.28	0.04	-0.21– 0.30
COVID-19 (year 2020)	-0.32	-0.81– 0.16	-0.32	-0.80– 0.16	-0.32	-0.80– 0.16
Constant	73.91	73.11– 74.70	73.93	73.13– 74.73	74.19	73.38– 75.00
R-squared within	0.039		0.040		0.041	

Table S4. Characteristics of the population in the supplementary analysis of self-rated health. SOEP, 1992-2021.[SD – standard deviation; N – number of cases; Min – minimum; Max – maximum.]

Variable	Mean	(SD)	N	(%)	Min	Max
Time-varying variables (N= 247,191 observations)						
Good or very good health (coded as 0 vs. 100)			139,331	(56.37)	0	100
Job insecurity:						
Current			101,504	(41.06)	0	1
Cumulative exposure	3.85	(4.42)			0	30
Recovery year 1			27,327	(11.06)	0	1
Recovery year 2			15,128	(6.12)	0	1
Recovery year 3			9,855	(3.99)	0	1
Unemployment:						
Current			13,968	(5.65)	0	1
Cumulative exposure	0.51	(1.49)			0	25
Recovery year 1			5,951	(2.41)	0	1
Recovery year 2			4,408	(1.78)	0	1
Recovery year 3			3,676	(1.49)	0	1
Inactivity:						
Current			26,218	(10.61)	0	1
Cumulative exposure	1.03	(2.42)			0	30
Recovery year 1			7,794	(3.15)	0	1
Recovery year 2			6,580	(2.66)	0	1
Recovery year 3			5,738	(2.32)	0	1
Age	43.30	(9.73)			18	65
Before Hartz reforms (before 2005)			89,431	(36.18)	0	1
Recession 1992-3			4,527	(1.81)	0	1
Recession 2002-3			29,688	(11.84)	0	1
Recession 2009-10			19,599	(7.82)	0	1
COVID-19 (year 2020)			11,965	(4.84)	0	1
Reference years			181,412	(73.39)	0	1
Time-invariant variables (n = 17,110 respondents)						
Years of schooling	12.61	(2.70)			7	18
Woman			9,300	(54.35)	0	1
Birth year (cohort)	1964.83	(10.75)			1936	1994

Table S5. Distribution of cumulative exposure to job insecurity, unemployment, and inactivity in the sample used in the supplementary analysis of self-rated health. SOEP, 1992-2021, n = 17,110 respondents.

	Overall	Job insecurity Severe ("very worried")	Mild ("somewhat worried")	Unemployment	Inactivity
Percent who experienced none	16.97%	53.77%	19.60%	74.61%	62.96%
Accumulated exposure - 25th percentile	1	0	1	0	0
Accumulated exposure - median	5	0	3	0	0
Accumulated exposure - 75th percentile	9	2	7	1	2
Accumulated exposure - 95th percentile	17	7	14	5	9
Accumulated exposure - 99th percentile	23	12	19	11	16

Table S6. Short- and long-term effects of job insecurity on the probability of 'good' or 'very good' self-rated health. Linear probability models with individual fixed effects and standard errors clustered on individuals. SOEP, 1992-2021, N= 247,191 observations and n = 17,110 respondents.[B – unstandardized coefficients, CI – confidence intervals]

	'Good' or 'very good' self-rated health	
	B[a]	95% CI
Job insecurity:		
Current	-3.00	-3.69– -2.30
Cumulative exposure	-0.86	-1.12– -0.61
Cumulative exposure squared	0.04	0.02– 0.05
Recovery year 1	-0.20	-0.91– 0.51
Recovery year 2	-0.14	-0.93– 0.65
Recovery year 3	-0.15	-1.02– 0.72
Unemployment:		
Current	-4.30	-5.53– -3.07
Cumulative exposure	-0.44	-1.06– 0.17
Cumulative exposure squared	0.01	-0.04– 0.06
Recovery year 1	-0.35	-1.65– 0.96
Recovery year 2	-0.71	-2.06– 0.64
Recovery year 3	-0.07	-1.47– 1.33
Inactivity:		
Current	-1.18	-2.20– -0.16
Cumulative exposure	-0.26	-0.66– 0.15
Cumulative exposure squared	0.01	-0.01– 0.03
Recovery year 1	-1.01	-2.13– 0.11
Recovery year 2	-1.42	-2.53– -0.31
Recovery year 3	-0.12	-1.23– 0.99
Age (centered at 40, per 10y)	-10.35	-11.59– -9.11
Age x Age	-1.52	-2.12– -0.92
Age x Years of schooling (centered at 12y)	0.56	0.39– 0.74
Age x Woman	1.47	0.51– 2.43
Age x Cohort (centered at 1965, per 10y)	-1.49	-2.59– -0.38
Job change	1.81	1.22– 2.40
Before Hartz reforms (before 2005)	2.16	1.32– 3.01
Recession 1992-3	8.34	6.87– 9.80
Recession 2001-3	-1.33	-1.94– -0.72
Recession 2009-10	0.23	-0.38– 0.83
COVID-19 (years 2020-2021)	4.03	3.06– 5.00
Constant	62.92	61.96– 63.88
R-squared within	0.035	

[a] The dichotomous variable has been rescaled to 0-100, to facilitate comparability of effect sizes with the main analysis.

Table S7. Short- and long-term effects of severe and mild job insecurity on mental and physical health (0-100). Models 1 and 2: fixed effect estimation with standard errors clustered on individuals. SOEP, 2002-2020, N= 84,219 observations and n = 12,624 respondents. Model 3: linear probability models with individual fixed effects and standard errors clustered on individuals. SOEP, 1992-2021, N= 24,7191 observations and n = 17,110 respondents.[B – unstandardized coefficients, CI – confidence intervals]

	Model 1 Mental health (0-100)		Model 2 Physical health (0-100)		Model 3 'Good'/'very good' self-rated health	
	B	95% CI	B	95% CI	B[a]	95% CI
Job insecurity						
Current – severe	-3.35	-3.81– -2.88	-2.58	-3.07– -2.09	-5.27	-6.18– -4.36
Current – mild	-1.62	-1.94– -1.30	-1.37	-1.72– -1.02	-2.71	-3.41– -2.00
Cumulative exposure – severe	-0.48	-0.73– -0.23	-0.64	-0.91– -0.37	-1.04	-1.47– -0.61
Cumulative exposure – mild	-0.41	-0.55– -0.26	-0.45	-0.61– -0.29	-0.66	-0.97– -0.36
Cumulative exposure squared – severe	0.02	-0.01– 0.04	0.03	0.00– 0.06	0.06	0.03– 0.09
Cumulative exposure squared – mild	0.03	0.02– 0.04	0.03	0.02– 0.04	0.04	0.02– 0.05
Recovery year 1	-0.09	-0.43– 0.26	-0.14	-0.51– 0.24	-0.35	-1.06– 0.36
Recovery year 2	-0.17	-0.54– 0.20	-0.12	-0.53– 0.29	-0.27	-1.06– 0.52
Recovery year 3	0.12	-0.31– 0.56	0.15	-0.32– 0.62	-0.25	-1.12– 0.62
Unemployment:						
Current	-4.86	-5.57– -4.14	-3.36	-4.12– -2.60	-4.61	-5.85– -3.38
Cumulative exposure	-0.55	-0.91– -0.18	-0.87	-1.24– -0.49	-0.40	-1.02– 0.21
Cumulative exposure squared	0.01	-0.02– 0.05	0.03	-0.00– 0.06	0.01	-0.04– 0.06
Recovery year 1	0.12	-0.63– 0.87	0.34	-0.45– 1.12	-0.23	-1.53– 1.07
Recovery year 2	0.05	-0.71– 0.81	0.30	-0.51– 1.12	-0.67	-2.02– 0.68
Recovery year 3	-0.09	-0.90– 0.71	0.70	-0.14– 1.55	-0.09	-1.49– 1.31
Inactivity:						
Current	-2.24	-2.76– -1.73	-2.22	-2.79– -1.66	-1.32	-2.35– -0.30
Cumulative exposure	-0.17	-0.41– 0.08	-0.02	-0.28– 0.24	-0.23	-0.64– 0.17
Cumulative exposure squared	0.00	-0.02– 0.02	-0.00	-0.02– 0.02	0.01	-0.01– 0.03
Recovery year 1	0.02	-0.56– 0.61	-0.39	-1.02– 0.24	-1.00	-2.12– 0.12
Recovery year 2	-0.75	-1.32– -0.19	-0.67	-1.26– -0.07	-1.40	-2.51– -0.29
Recovery year 3	-0.76	-1.39– -0.13	-0.11	-0.74– 0.51	-0.10	-1.21– 1.01
Age (centered at 40, per 10y)	-3.40	-4.51– -2.29	-6.13	-7.35– -4.92	-10.61	-11.85– -9.37
Age x Age	1.46	0.77– 2.15	1.04	0.28– 1.80	-1.51	-2.11– -0.91
Age x Years of schooling (centered at 12y)	0.17	0.09– 0.25	0.37	0.29– 0.46	0.54	0.36– 0.72
Age x Woman	-0.30	-0.76– 0.16	0.08	-0.41– 0.58	1.43	0.47– 2.39
Age x Cohort (centered at 1965, per 10y)	3.20	1.85– 4.55	3.07	1.59– 4.56	-1.57	-2.67– -0.47
Job change	0.94	0.56– 1.31	1.16	0.75– 1.58	1.87	1.28– 2.46
Recession 1992-3	—		—		8.50	7.04– 9.97
Before Hartz reforms (before 2005)	-0.63	-1.06– -0.20	-0.54	-0.99– -0.08	2.15	1.30– 2.99
Recession 2001-3	-1.87	-2.30– -1.44	-2.12	-2.58– -1.67	-1.37	-1.99– -0.76
Recession 2009-10	-0.02	-0.24– 0.21	0.07	-0.18– 0.32	0.26	-0.34– 0.86
COVID-19	-1.63	-2.07– -1.18	-0.30	-0.79– 0.18	4.09	3.12– 5.06
Constant	73.66	72.91– 74.40	74.29	73.49– 75.10	62.84	61.89– 63.79
R-squared within	0.020		0.042		0.035	

[a] The dichotomous variable has been rescaled to 0-100, to facilitate comparability of effect sizes with the main analysis.

Table S8. Short- and long-term effects of job insecurity on mental health (0-100) of men and women. SOEP, 2002-2020, Men: N= 37,553 observations and n = 5,620 respondents. Women: N= 46,666 observations and n = 7,004 respondents. [B – unstandardized coefficients, CI – confidence intervals]

	Men		Women	
	B	95% CI	B	95% CI
Job insecurity:				
Current	-1.99	-2.44– -1.54	-1.86	-2.30– -1.41
Cumulative exposure	-0.33	-0.51– -0.15	-0.43	-0.63– -0.23
Cumulative exposure squared	0.01	0.00– 0.02	0.02	0.01– 0.03
Recovery year 1	-0.13	-0.63– 0.37	0.03	-0.45– 0.51
Recovery year 2	0.04	-0.48– 0.56	-0.29	-0.80– 0.23
Recovery year 3	-0.15	-0.77– 0.47	0.38	-0.21– 0.98
Unemployment:				
Current	-5.00	-6.09– -3.91	-4.44	-5.39– -3.50
Cumulative exposure	-0.49	-1.00– 0.01	-0.56	-1.08– -0.04
Cumulative exposure squared	0.01	-0.04– 0.05	0.02	-0.03– 0.07
Recovery year 1	0.07	-1.05– 1.19	0.18	-0.83– 1.18
Recovery year 2	-0.09	-1.20– 1.02	0.18	-0.85– 1.22
Recovery year 3	0.31	-0.93– 1.54	-0.29	-1.36– 0.78
Inactivity:				
Current	-3.95	-5.31– -2.59	-1.81	-2.39– -1.24
Cumulative exposure	-0.80	-1.50– -0.09	-0.09	-0.36– 0.18
Cumulative exposure squared	0.06	0.00– 0.11	-0.00	-0.02– 0.02
Recovery year 1	-0.82	-2.23– 0.59	0.35	-0.29– 1.00
Recovery year 2	-1.20	-2.48– 0.07	-0.57	-1.19– 0.06
Recovery year 3	-1.45	-2.83– -0.07	-0.44	-1.15– 0.27
Age (centered at 40, per 10y)	-4.39	-5.94– -2.84	-2.85	-4.37– -1.32
Age x Age	2.28	1.30– 3.26	0.85	-0.12– 1.82
Age x Years of schooling (centered at 12y)	0.16	0.05– 0.27	0.20	0.08– 0.32
Age x Cohort (centered at 1965, per 10y)	4.81	2.88– 6.74	2.01	0.13– 3.89
Job change	1.45	0.92– 1.99	0.61	0.09– 1.13
Before Hartz reforms (before 2005)	-0.72	-1.33– -0.12	-0.55	-1.16– 0.06
Recession 2001-3	-1.79	-2.40– -1.18	-1.75	-2.36– -1.14
Recession 2009-10	-0.24	-0.56– 0.08	0.12	-0.20– 0.43
COVID-19 (2020)	-1.86	-2.49– -1.23	-1.48	-2.10– -0.86
Constant	75.68	74.58– 76.78	71.70	70.69– 72.71
R-squared within	0.022		0.017	

Table S9. Short- and long-term effects of job insecurity on physical health (0-100) of men and women. SOEP, 2002-2020, Men: N= 37,553 observations and n = 5,620 respondents. Women: N= 46,666 observations and n = 7,004 respondents. [B – unstandardized coefficients, CI – confidence intervals]

	Men		Women	
	B	95% CI	B	95% CI
Job insecurity:				
Current	-1.86	-2.36– -1.35	-1.33	-1.80– -0.86
Cumulative exposure	-0.39	-0.59– -0.19	-0.54	-0.75– -0.33
Cumulative exposure squared	0.01	0.00– 0.03	0.02	0.01– 0.04
Recovery year 1	-0.36	-0.92– 0.20	0.14	-0.37– 0.66
Recovery year 2	-0.03	-0.62– 0.57	-0.12	-0.69– 0.44
Recovery year 3	0.30	-0.39– 0.99	0.11	-0.52– 0.75
Unemployment:				
Current	-3.54	-4.73– -2.35	-3.06	-4.05– -2.06
Cumulative exposure	-0.88	-1.46– -0.30	-0.86	-1.36– -0.37
Cumulative exposure squared	0.02	-0.03– 0.07	0.03	-0.01– 0.08
Recovery year 1	0.16	-1.05– 1.38	0.56	-0.47– 1.59
Recovery year 2	1.04	-0.15– 2.22	-0.16	-1.26– 0.95
Recovery year 3	0.67	-0.61– 1.95	0.83	-0.30– 1.96
Inactivity:				
Current	-4.48	-5.98– -2.98	-1.75	-2.37– -1.13
Cumulative exposure	-0.65	-1.45– 0.15	0.09	-0.19– 0.37
Cumulative exposure squared	0.04	-0.04– 0.11	-0.01	-0.03– 0.01
Recovery year 1	-1.10	-2.56– 0.36	-0.08	-0.79– 0.63
Recovery year 2	-0.73	-2.01– 0.56	-0.59	-1.26– 0.08
Recovery year 3	-0.08	-1.40– 1.24	0.00	-0.70– 0.71
Age (centered at 40, per 10y)	-6.88	-8.60– -5.15	-5.35	-7.01– -3.68
Age x Age	1.65	0.54– 2.76	0.61	-0.44– 1.65
Age x Years of schooling (centered at 12y)	0.37	0.25– 0.50	0.40	0.28– 0.52
Age x Cohort (centered at 1965, per 10y)	4.36	2.19– 6.53	2.16	0.12– 4.20
Job change	1.85	1.24– 2.46	0.68	0.12– 1.24
Before Hartz reforms (before 2005)	-0.73	-1.38– -0.09	-0.34	-0.97– 0.29
Recession 2001-3	-1.84	-2.48– -1.20	-2.23	-2.86– -1.59
Recession 2009-10	-0.08	-0.44– 0.28	0.16	-0.19– 0.51
COVID-19 (2020)	-0.58	-1.29– 0.12	-0.14	-0.80– 0.52
Constant	76.40	75.18– 77.61	72.37	71.29– 73.44
R-squared within	0.050		0.036	

Table S10. Short- and long-term effects of job insecurity on mental health (0-100) of higher and lower educated. SOEP, 2002-2020, Higher educated: N= 42,001 observations and n = 6,230 respondents. Lower educated: N= 42,218 observations and n = 6,394 respondents. [B – unstandardized coefficients, CI – confidence intervals]

	Higher educated (12+ years of schooling)		Lower educated (<12 years of schooling)	
	B	95% CI	B	95% CI
Job insecurity:				
Current	-1.47	-1.90– -1.04	-2.40	-2.87– -1.92
Cumulative exposure	-0.39	-0.58– -0.20	-0.40	-0.59– -0.20
Cumulative exposure squared	0.02	0.00– 0.03	0.02	0.01– 0.03
Recovery year 1	0.12	-0.34– 0.59	-0.27	-0.78– 0.24
Recovery year 2	-0.27	-0.77– 0.23	-0.06	-0.60– 0.47
Recovery year 3	0.59	0.02– 1.16	-0.32	-0.97– 0.32
Unemployment:				
Current	-3.91	-5.15– -2.66	-5.26	-6.14– -4.38
Cumulative exposure	-0.15	-0.93– 0.64	-0.64	-1.06– -0.22
Cumulative exposure squared	-0.04	-0.12– 0.04	0.03	-0.01– 0.06
Recovery year 1	0.45	-0.84– 1.73	-0.16	-1.08– 0.76
Recovery year 2	0.07	-1.23– 1.37	-0.03	-0.97– 0.91
Recovery year 3	0.02	-1.34– 1.37	-0.20	-1.22– 0.81
Inactivity:				
Current	-0.85	-1.56– -0.15	-3.46	-4.20– -2.72
Cumulative exposure	-0.06	-0.44– 0.33	-0.23	-0.57– 0.10
Cumulative exposure squared	-0.00	-0.03– 0.03	0.01	-0.02– 0.03
Recovery year 1	0.27	-0.56– 1.09	-0.27	-1.10– 0.56
Recovery year 2	-0.11	-0.86– 0.65	-1.40	-2.23– -0.58
Recovery year 3	-0.40	-1.28– 0.47	-1.07	-1.97– -0.18
Age (centered at 40, per 10y)	-2.55	-4.10– -1.01	-3.06	-4.76– -1.36
Age x Age	1.46	0.53– 2.40	1.42	0.40– 2.45
Age x Years of schooling (centered at 12y)	0.04	-0.09– 0.17	0.66	0.25– 1.07
Age x Woman	-0.10	-0.71– 0.50	-0.67	-1.36– 0.02
Age x Cohort (centered at 1965, per 10y)	3.37	1.55– 5.18	2.94	0.93– 4.95
Job change	0.29	-0.23– 0.80	1.54	0.99– 2.08
Before Hartz reforms (before 2005)	-0.46	-1.07– 0.15	-0.78	-1.39– -0.17
Recession 2001-3	-1.34	-1.95– -0.74	-2.16	-2.77– -1.55
Recession 2009-10	-0.02	-0.33– 0.29	-0.07	-0.40– 0.26
COVID-19 (2020)	-1.77	-2.35– -1.19	-1.52	-2.21– -0.83
Constant	73.57	72.59– 74.55	73.55	72.42– 74.68
R-squared within	0.010		0.028	

Table S11. Short- and long-term effects of job insecurity on physical health (0-100) of higher and lower educated. SOEP, 2002-2020, Higher educated: N= 42,001 observations and n = 6,230 respondents. Lower educated: N= 42,218 observations and n = 6,394 respondents. [B – unstandardized coefficients, CI – confidence intervals]

	Higher educated (12+ years of schooling)		Lower educated (<12 years of schooling)	
	B	95% CI	B	95% CI
Job insecurity:				
Current	-1.04	-1.49– -0.59	-2.09	-2.61– -1.56
Cumulative exposure	-0.36	-0.55– -0.17	-0.61	-0.83– -0.39
Cumulative exposure squared	0.02	0.01– 0.03	0.02	0.01– 0.04
Recovery year 1	-0.04	-0.54– 0.46	-0.19	-0.75– 0.37
Recovery year 2	-0.17	-0.72– 0.39	-0.04	-0.64– 0.56
Recovery year 3	0.27	-0.35– 0.90	0.04	-0.65– 0.74
Unemployment:				
Current	-1.88	-3.15– -0.61	-4.04	-5.00– -3.08
Cumulative exposure	-0.65	-1.45– 0.14	-0.95	-1.39– -0.52
Cumulative exposure squared	-0.00	-0.09– 0.08	0.04	0.00– 0.08
Recovery year 1	0.90	-0.36– 2.15	-0.03	-1.03– 0.96
Recovery year 2	-0.59	-1.88– 0.70	0.60	-0.42– 1.63
Recovery year 3	0.93	-0.44– 2.30	0.54	-0.54– 1.61
Inactivity:				
Current	-1.29	-2.05– -0.53	-3.09	-3.92– -2.26
Cumulative exposure	0.16	-0.22– 0.54	-0.19	-0.55– 0.16
Cumulative exposure squared	-0.01	-0.04– 0.02	0.00	-0.02– 0.03
Recovery year 1	-0.64	-1.51– 0.23	-0.25	-1.17– 0.67
Recovery year 2	-0.19	-0.97– 0.59	-1.17	-2.07– -0.27
Recovery year 3	0.04	-0.80– 0.88	-0.23	-1.15– 0.68
Age (centered at 40, per 10y)	-5.10	-6.74– -3.46	-5.57	-7.49– -3.65
Age x Age	0.88	-0.13– 1.89	1.19	0.05– 2.33
Age x Years of schooling (centered at 12y)	0.21	0.08– 0.35	0.98	0.51– 1.46
Age x Woman	0.07	-0.58– 0.71	-0.11	-0.88– 0.66
Age x Cohort (centered at 1965, per 10y)	2.80	0.83– 4.78	3.27	1.04– 5.50
Job change	0.45	-0.10– 1.01	1.80	1.19– 2.41
Before Hartz reforms (before 2005)	-0.16	-0.79– 0.47	-0.90	-1.55– -0.25
Recession 2001-3	-1.33	-1.95– -0.70	-2.72	-3.38– -2.07
Recession 2009-10	-0.22	-0.57– 0.13	0.31	-0.05– 0.68
COVID-19 (2020)	-0.12	-0.74– 0.51	-0.56	-1.30– 0.18
Constant	75.04	73.99– 76.09	73.54	72.31– 74.78
R-squared within	0.025		0.057	

Table S12. Effects' sizes predicted on the basis of the main analysis (as presented in Table 2)

	Effect on mental health		Effect on physical health	
	B	95% CI	B	95% CI
1. Predicted long term effect of a single exposure to job insecurity (including both linear and quadratic effects of accumulated exposure)	-0.37<br>19.5% of the short-term effect<br>112% of the average annual decline	-0.50– -0.25	-0.45<br>28.9% of the short-term effect<br>75% of the average annual decline	-0.59– -0.32
2. Predicted long-term effect of four exposures to job insecurity	-1.30<br>67.7% of the short-term effect<br>390% of the average annual decline	-1.73– -0.87	-1.58<br>100.5% of the short-term effect<br>261% of the average annual decline	-2.04– -1.11
3. Predicted long-term effect of 14 exposures to job insecurity	-2.21<br>115.2% of the short-term effect<br>664% of the average annual decline	-3.14– -1.27	-2.74<br>174.9% of the short-term effect<br>454% of the average annual decline	-3.75– -1.73
4. Predicted rate of baseline average annual health decline[a]	-0.33	-0.44– -0.22	-0.60	-0.73– -0.48

[a] The rate of baseline average annual health decline due to aging was not linear; it changed with age. The predictions presented in this Table refer to the average rate of decline between the age of 30 and 50.
